# Supplementary material for: Heparanase, a host gene that potently restricts retrovirus transcription
Source: mBio. 2025 Feb 25;16(4):e03252-24. doi: 10.1128/mbio.03252-24 (PMC11980367; doi:10.1128/mbio.03252-24)
Supplement: Supplemental material — Figs. S1 to S8; Table S1. [file mbio.03252-24-s0001.pdf]

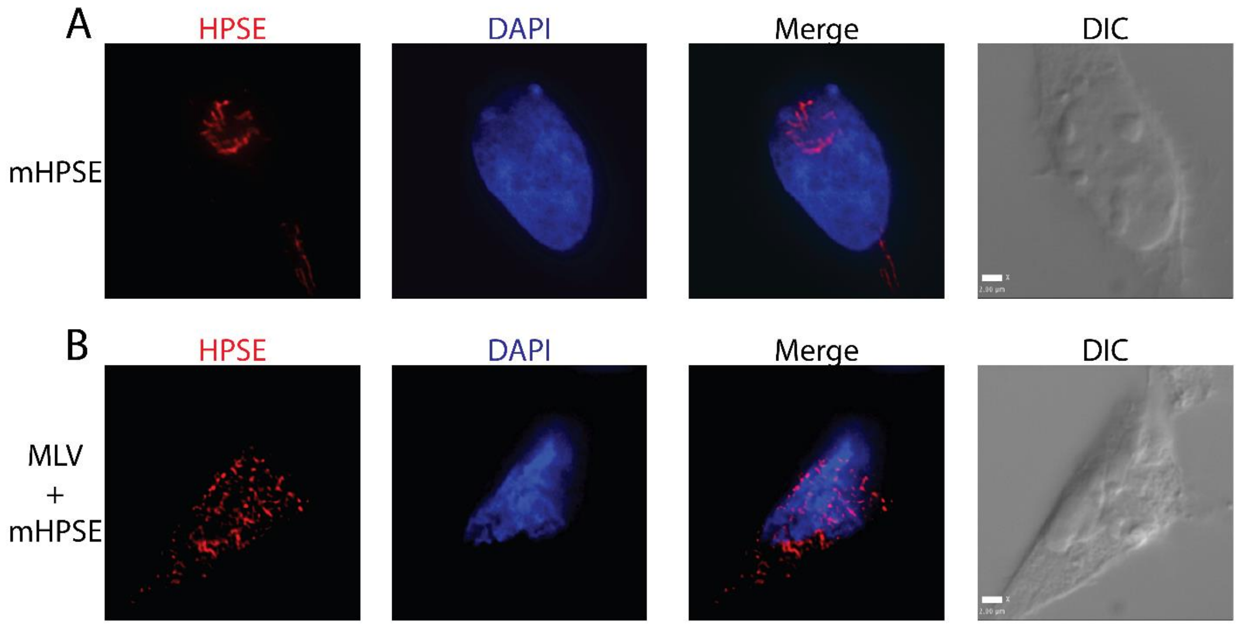

Fig S1. mHPSE is primarily localized in the nucleus. AD-293 cells were transfected with a plasmid encoding **(A)** mHPSE or **(B)** co-transfected with an MLV infectious clone and a plasmid encoding mHPSE and visualized by immunostaining. Images were acquired using 100×/1.46 Plan Apo oil immersion objective on a motorized Zeiss Axioimager M2 microscope. Scale bar = 2 µm. Representative deconvolved single Z-section images are shown. (N=2)

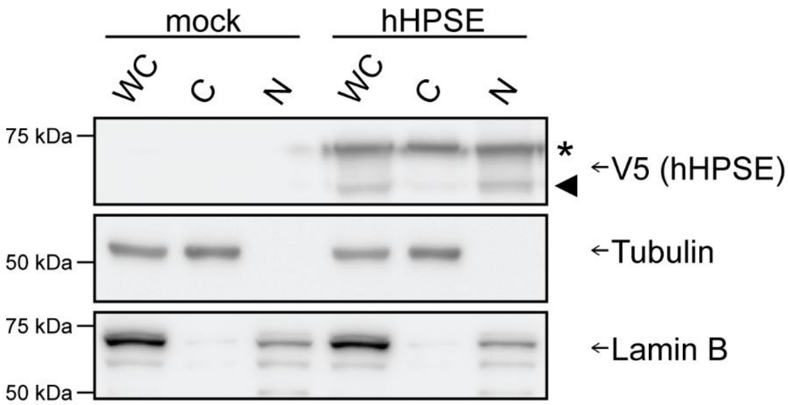

Fig S2. hHPSE is present in the nucleus. Immunobloting of whole cell (WC), cytosolic (C), or nuclear (N) fractions of 293T cells transfected with a plasmid encoding hHPSE. Samples were analyzed for the indicated proteins. Representative immunoblotting results of 3 independent experiments are shown.

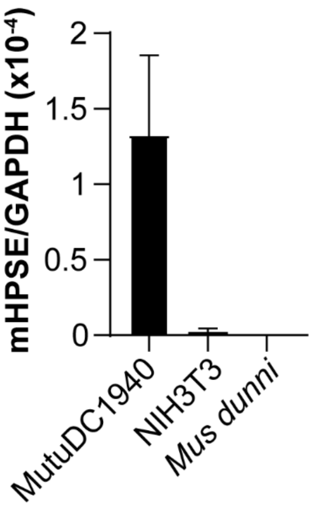

Fig S3. Mouse *Hpse* is not expressed in NIH3T3 or *Mus dunni* cells. RNA was isolated from MutuDC1940, NIH3T3, and *Mus dunni* cells, followed by RT-qPCR to evaluate *Hpse* expression levels normalized to *Gapdh*. (N=3)

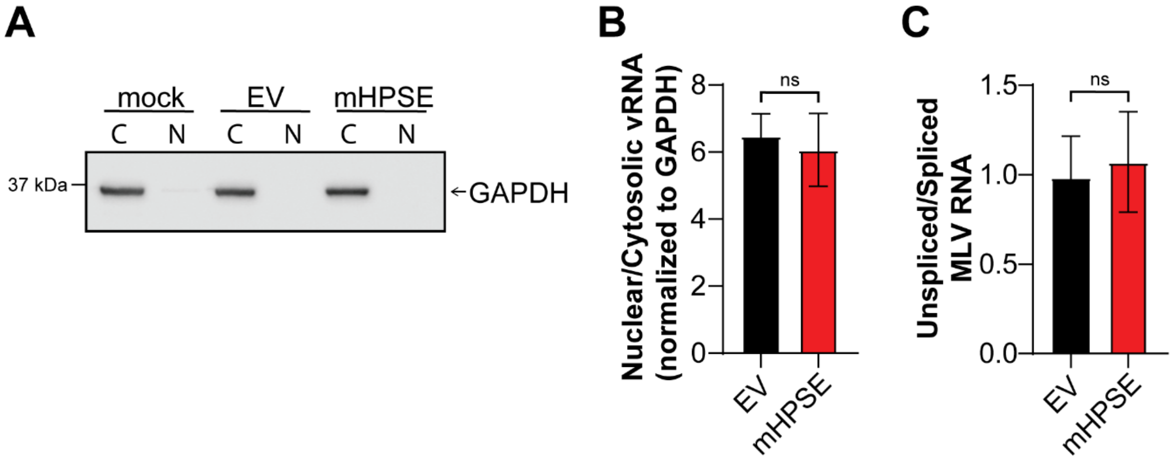

Fig S4. Mouse heparanase (mHPSE) does not affect the export or splicing of MLV RNA. 293T cells were co-transfected with plasmids encoding an MLV infectious clone and either mHPSE or empty vector (EV). **(A-B)** mHPSE does not affect MLV RNA export. Following co-transfection, samples were fractionated into cytosolic (C) and nuclear (N) fractions. **(A)** Purity of nuclear fractions was analyzed by immunoblotting for the cytosolic protein, GAPDH. Representative western blot of 3 independent experiments is shown. **(B)** MLV RNA transcripts in C and N fractions were quantified by RT-qPCR. Data presented as the ratio of MLV RNA in N/C fractions in the presence of mHPSE or EV and normalized to *GADPH*. **(C)** mHPSE does not affect the splicing of MLV RNA. Following co-transfection, levels of spliced and unspliced MLV RNA were determined by RT-PCR and the ratio of unspliced/spliced MLV RNA was calculated. All data in graphs presented as mean ± SEM. Statistical analysis was performed by unpaired t test, N=3, ns; not significant.

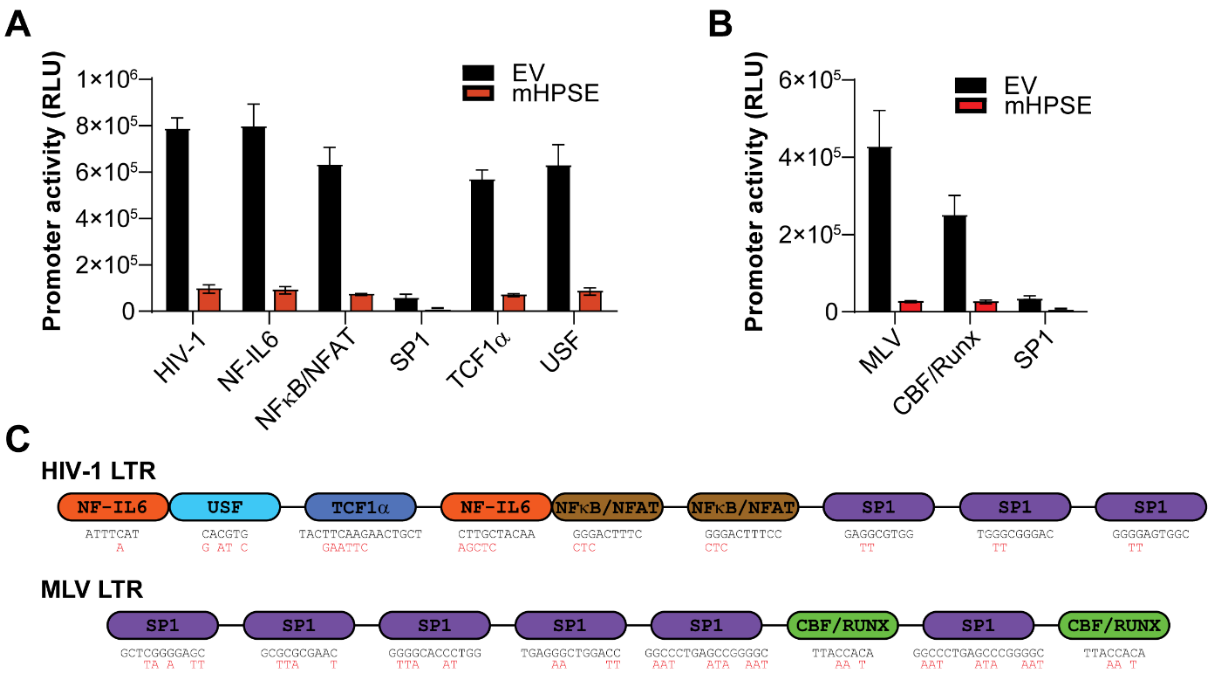

Fig S5. Mutations in SP1 binding sites reduce retrovirus LTR activity. 293T cells were co-transfected with **(A)** HIV-1 or **(B)** MLV LTR luciferase reporter constructs containing mutations at the indicated transcription factor binding site along with plasmids expressing either EV or mouse heparanase (mHPSE). Data presented as mean ± SEM, **(A)** N=4 and **(B)** N=3, RLU; relative light units. **(C)** Diagram displaying the mutations made in the-various transcription factor binding sites of the MLV and HIV-1 LTRs.

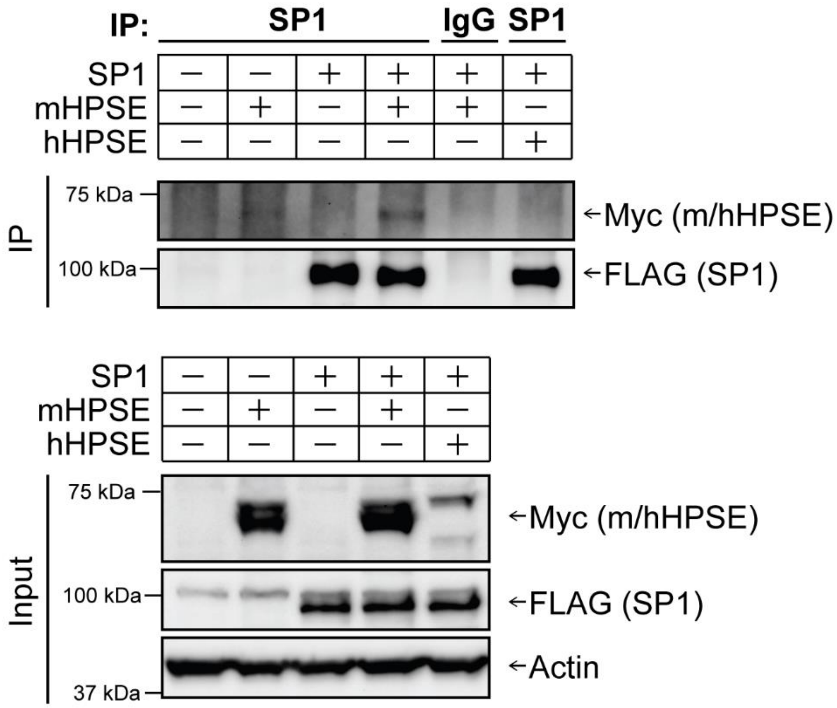

Fig S6. mHPSE associates with SP1. Immunoblots of 293T cells transfected with plasmids encoding either empty vector (EV), mHPSE, or SP1, or co-transfected with plasmids expressing SP1 and either mHPSE or human heparanase (hHPSE). Co-immunoprecipitation (coIP) was then performed using an anti-SP1 antibody or an isotype control (IgG). Samples were analyzed by immunoblotting for the indicated proteins and shown here is a representative of 2 independent experiments.

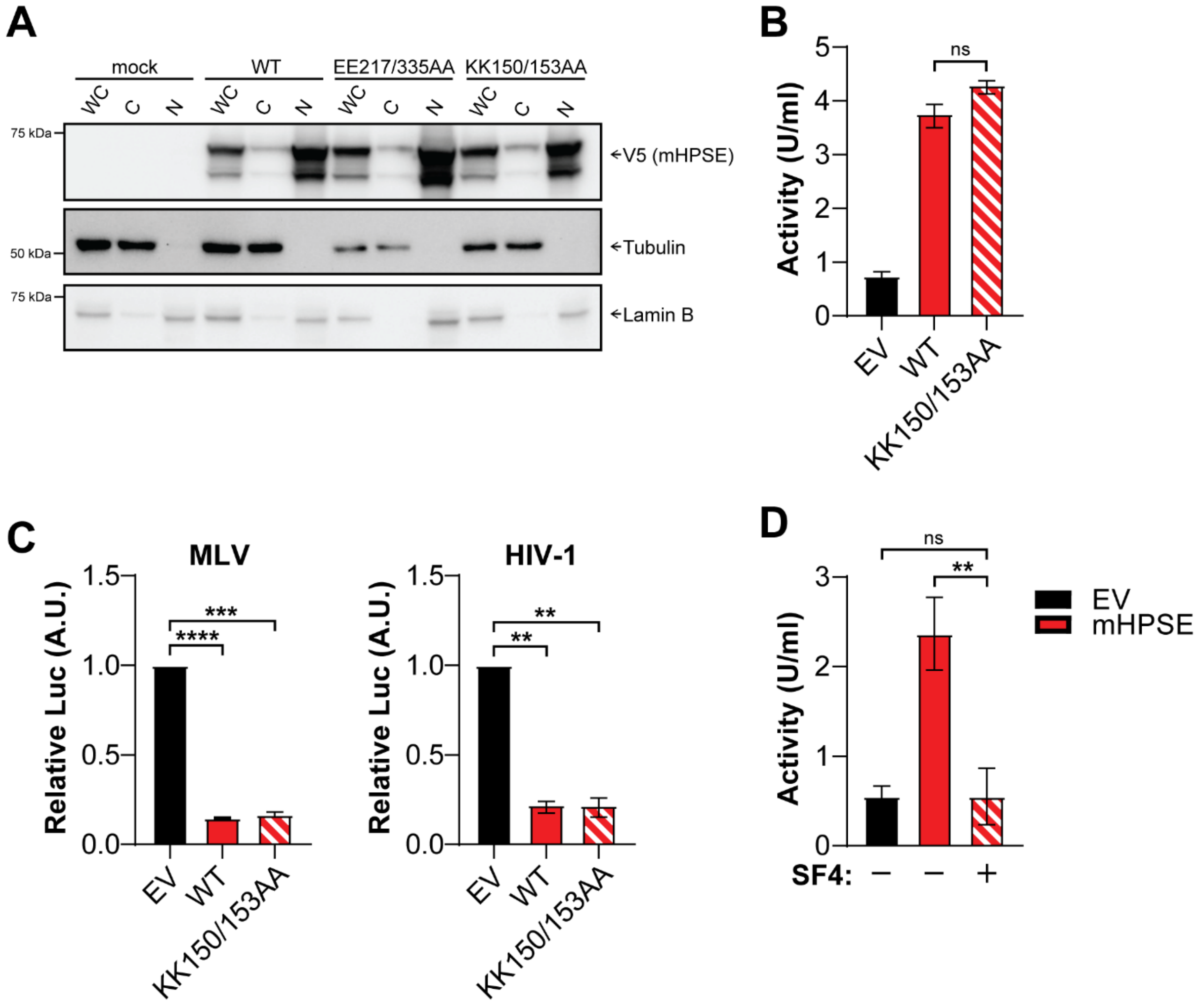

Fig S7. KK150/153 of mouse heparanase (mHPSE) do not affect its antiretroviral function

(A) mHPSE (WT) glutamic acid (E) residues 217 and 335 and lysine (K) residues 150 and 153 do not affect mHPSE nuclear localization. Cells transfected with the indicated plasmids were fractionated into whole cell lysate (WC), cytosolic fraction (C), or nuclear fraction (N) followed by immunoblotting. Immunoblots shown are representative of 3 independent experiments. (**B**) K residues 150 and 153 do not affect the enzymatic activity of mHPSE. 293T cells were transfected with plasmids encoding either empty vector (EV), wild type mHPSE (WT), or mHPSE in which either K residues 150 and 153 are substituted for alanine (KK150/153AA) followed by measurement of mHPSE enzymatic activity. **(C)** Lysine residues 150 and 153 are dispensable to

the antiretroviral function of mHPSE. 293T cells were co-transfected with either MLV LTR or HIV-1 LTR luciferase reporter constructs and plasmids encoding EV, WT, or KK150/153AA followed by luciferase measurements. **(D)** SF4 inhibits the enzymatic activity of mHPSE. 293T cells were transfected with either EV or mHPSE and then treated with SF4 (100 μM) or vehicle control, followed by measurement of enzymatic activity. All data in graphs presented as mean ± SEM. Statistical analysis was performed by **(B)** unpaired t test, N=4, **(C)** one sample t test, N=3, or **(D)** one-way ANOVA, Dunnett's multiple comparison test, N=5, ns; not significant, $^{**}P \leq 0.01$, $^{***}P \leq 0.001$, $^{****}P \leq 0.0001$.

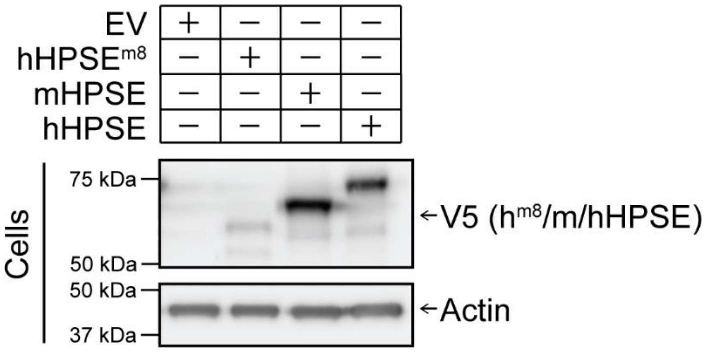

Fig S8. hHPSE^{m8} expresses at very low levels. 293T cells were transfected with plasmids encoding either empty vector (EV), mouse heparanase (mHPSE), human heparanase (hHPSE), or hHPSE^{m8}, and protein samples were analyzed by immunoblotting for the indicated proteins. Displayed immunoblots are representative of 3 independent experiments.

Table S1: Primers

Name of Primer	Nucleotide Sequence
mHPSEcloF	5’-GGAAGCTTACCATGCTGAGGCTG-3’
mHPSEcloR	5’-GTCTAGATATACAAGCAGCGATTTTGG-3’
MuHep_hu8KDF	5’-TCCCAAGAAGGAACCGACTTCCGAAGAAAGAAG-3’
MuHep_hu8KDR	5’- TCCACGACGTCCTGGGTCGGCGCGGTCCCCGCG-3’
hu8KD_m50F	5’-ACCGCGCCGACCCAGGACGTCGTGGACCTGGA-3’
hu8KD_m50R	5’-TCTTCGGAAGTCGGTTCCTTCTTGGGATCGAAAATTAGG-3’
Mu8KD_h50F	5’- CCTGCGCAAGCAGACGACGTGGTAGACTTGGAG-3’
Mu8KD_h50R	5’-TTCAAAGGTTGATTCCTTGTCCGGATCAAAAATAAGG-3’
HuHep_M8KDF	5’-CCGGACAAGGAATCAACCTTTGAAGAGAGAAG-3’
HuHep_M8KDR	5’-TACCACGTCGTCTGCTTGCGCAGGTCGGGGCA-3’
CXm50^{N}F	5’-GTTCTTGAACTTTTTTTGGTACTGCTCTCGGAG-3’
CXm50^{N}R	5’-GTGGAGAAGTGATTGACTCTCTTACATGGCATC-3’
CXh50^{N}F	5’-CGAGAGCAGTACCAAAAAAAGTTCAAGAACAGC-3’
CXh50^{N}R	5’-GCCATGTAAGAGAGTCAATCACTTCTCCACCAG-3’
CXm50^{M}F	5’-TCACTTCTCCACCAGCCTTCAGGAAACTCCTCA-3’
CXm50^{M}R	5’-AATGGCAAGCGTGCAAGGCCCAGACAGGAG-3’
CXh50^{M}F	5’-GGAGTTTCCTGAAGGCTGGTGGAGAAGTGATTG-3’
CXh50^{M}R	5’-CTCCTGTCTGGGCCTTGCACGCTTGCCATTAAC-3’
CXm50^{C}F	5’-ACGCTTGCCATTAACACCCTGGGACCTACC-3’
CXm50^{C}R	5’-GTTGCTGCTTGCATCTGAAAATAAAAGGCATACGGT-3’
CXh50^{C}F	5’-GGTAGGTCCCAGGGTGTTAATGGCAAGCGT-3’
CXh50^{C}R	5’-TGCCTTTTATTTTCAGATGCAAGCAGCAACTTTGGC-3’
mCdomainF	5’-CTGTTCAAGAAACTGGTAGGTCCCAGGGTG-3’
mCdomainR	5’-GGGCCCTCTAGATATACAAGCAGCGATTTT-3’
hΔCF	5’- GCTGCTTGTATATCTAGAGGGCCCGCG-3’
hΔCR	5’- GGGACCTACCAGTTTCTTGAACAGAAGAGA-3’
mHPSE_Myc_N_term_F	5’-AGCGAAGAAGATCTTGACGACGTGGTAGACTTGGAG-3’
mHPSE_Myc_N_term_R	5’-AATAAGTTTTTGTTCGGTCGGCGCGGT-3’
hHPSE_Myc_N_term_F	5’- AGCGAAGAAGATCTTCAGGACGTCGTGGACC-3’
hHPSE_Myc_N_term_R	5’- AATAAGTTTTTGTTCTGCTTGCGCAGGTCGG-3’
E335F	5’-GTTGGGAGcGACGAGCTC-3’
E335R	5’-CAGACCTTCTTGCCAGGTG-3’
E217F	5’-TGGGCAATGCGCCCAACA-3’
E217R	5’-GTTCCCAGGAGATGTTATAACCCTTGG-3’
KK150/153AA_F	5-GTACCAAGCGGAGTTCGCGAACAGC-3’
KK150/153AA_R	5’-TGCTCTCGGAGCAGCAACAGCT-3’
pSV-FMLV-Vec-F	5’-GAGCCACAATAAATAAAATAAAAGATTTTATTTAGTCTCCAG-3’
pSV-FMLV-Vec-R	5’-TGAACACGCCATGTCCGTCCAGAGGATGGT-3’
pSV-FMLV-env-F	5’-CCTCTGGACGGACATGGCGTGTTCAACGCTC-3’
pSV-FMLV-env-R	5’-CTTTTATTTTATTTATTGTGGCTCGTATTCTAGTGG-3’
